# Impact of the pulse contrast ratio on molybdenum K_α_ generation by ultrahigh intensity femtosecond laser solid interaction

**DOI:** 10.1038/s41598-018-22487-3

**Published:** 2018-03-07

**Authors:** Y. Azamoum, V. Tcheremiskine, R. Clady, A. Ferré, L. Charmasson, O. Utéza, M. Sentis

**Affiliations:** Aix Marseille Université, CNRS, LP3 UMR 7341, 13288 Marseille, France

## Abstract

We present an extended experimental study of the absolute yield of K_α_ x-ray source (17.48 keV) produced by interaction of an ultrahigh intensity femtosecond laser with solid Mo target for temporal contrast ratios in the range of 1.7 × 10^7^–3.3 × 10^9^ and on three decades of intensity 10^16^–10^19^  W/cm². We demonstrate that for intensity I ≥ 2 × 10^18^  W/cm² K_α_ x-ray emission is independent of the value of contrast ratio. In addition, no saturation of the K_α_ photon number is measured and a value of ~2 × 10^10^ photons/sr/s is obtained at 10 Hz and I ~10^19^  W/cm². Furthermore, K_α_ energy conversion efficiency reaches the same high plateau equal to ~2 × 10^−4^ at I = 10^19^  W/cm² for all the studied contrast ratios. This original result suggests that relativistic J × B heating becomes dominant in these operating conditions which is supposed to be insensitive to the electron density gradient scale length L/λ. Finally, an additional experimental study performed by changing the angle of incidence of the laser beam onto the solid target highlights a clear signature of the interplay between collisionless absorption mechanisms depending on the contrast ratio and intensity.

## Introduction

Optimization of ultrafast x-ray emission from plasmas produced by femtosecond high intensity laser-solid interaction is today under strong investigation by many groups around the world^1,2^. The interest towards developing intense and compact ultrashort x-ray sources is motivated by important scientific applications like time-resolved x-ray diffraction^3,4^ or x-ray absorption spectroscopy for advanced high-resolution diagnostics of materials driven to extreme thermodynamic conditions^5^. Furthermore, these sources provide powerful tools in societal applications such as phase contrast imaging for developing highly resolved diagnostics for biology^6^ and medicine^7^.

Laser-driven ultrashort hard x-ray pulses based on K_α_ radiation from solid targets are studied since decades^8,9^. Nevertheless, the constant improvement of femtosecond laser performances^10^ enables to explore a wide range of regimes of laser interaction where K_α_ emission can be strongly enhanced. In fact, laser pulses of intensities >10^18^ W/cm² and pulse duration <30 fs with controlled high contrast ratios (>10^8^) are today routinely obtained in laboratory using a single laser source with moderate peak power (~10 TW).

K_α_ x-ray emission is induced by the produced hot electrons due to the laser energy absorption by the plasma formed during the interaction. Therefore, K_α_ spectral lines radiation is governed by the energy distribution of the hot electron population accelerated by the laser electric field and/or the laser-induced plasma field. Key parameters to generate efficient hot electrons for K_α_ line production are mainly the laser electric field (i. e. intensity) and the fraction of the pulse energy converted into hot electrons. This fraction depends on the laser energy coupling to the plasma. The latter is driven by several collisionless absorption mechanisms which relative importance is controlled by the laser pulse parameters such as intensity, duration, temporal contrast ratio, angle of incidence, polarization, *etc*.^9,11–15^. For an obliquely incident p-polarized pulse, the laser energy absorption also relies on the electron density gradient scale length L of the plasma formed on the target before the main pulse. L is defined as ${\rm{L}}={[\frac{1}{{{\rm{n}}}_{{\rm{e}}}}(\frac{{{\rm{dn}}}_{{\rm{e}}}}{{\rm{dx}}})]}^{-1}$, *n*_*e*_ being the electron density and *x* the axis in the direction normal to the target surface. The parameter L is strongly dependent on the temporal contrast ratio CR, defined by ${C}{\rm{R}}={{\rm{I}}}_{{\rm{peak}}}/{{\rm{I}}}_{{\rm{background}}}$. CR is the ratio between the peak pulse intensity I_peak_ and the intensity of the background I_background_ present before the main pulse. The background consists of nanosecond amplified spontaneous emission (ASE) pedestal or any pre-pulses. If any background component is intense enough, a pre-plasma can develop in front of the solid target, modifying the laser absorption conditions and accordingly the K_α_ yield. For L > 0.1 λ and for low intensity I × λ² < 10^18^ W µm²/cm² (λ in µm,) resonance absorption prevails over other absorption mechanisms^12,16^. For L < 0.1 λ vacuum heating becomes the dominant mechanism^11,12,17^. For high intensity I × λ² > 10^18^ W µm²/cm², the magnetic component of the Lorentz force applied to the electron, negligible at low intensity, becomes comparable to the electric component so that relativistic J × B heating is supposed to become significant for K_α_ generation^18,19^.

Over the past two decades, most of the studies of K_α_ generation at λ = 800 nm were performed at low contrast ratio (~10^5^–10^6^) thus in conditions where an extended plasma was formed and had developed before the main pulse. In these operating conditions and for high Z element (Ag, Z = 47, Mo, Z = 42), the maximum of K_α_ conversion efficiency was observed at ~4 × 10^−5^ in 2π steradians (sr)^9,20,21^. With further improvement of the contrast ratio (~10^8^) at the fundamental wavelength (800 nm) as reported by Bastiani *et al*.^22^ and Lu *et al*.^23^, the optimization of the energy coupling to the target and thus the enhancement of the K_α_ yield was obtained using controlled pre-pulses. Even with such a high contrast ratio (~10^8^), a pre-plasma was formed on the target before the main pulse and resonance absorption was identified as the main absorption mechanism. In order to optimize the energy absorption, the aim was to generate experimentally the suitable plasma scale length L to improve the fraction of the absorbed laser energy by the plasma by resonance absorption. In these conditions, the optimal plasma scale length is determined in correspondence with a specific angle of incidence^16^. Furthermore, based on the optimal scale length for resonance absorption and the numerical study by Reich *et al*.^14^, a scaling law was established defining an optimum intensity I_opt_ to enhance K_α_ emission yield for a given target material, I_opt_ = 7 × 10^9^ × Z^4.4^ W/cm². The optimal intensity for Mo element (Z = 42) is I_opt_ ~ 10^17^ W/cm². It was also predicted a saturation or even a reduction of the K_α_ yield for intensities higher than the calculated optimal intensity. Moreover, many studies showed that a cleaner laser pulse characterized by a high temporal contrast ratio (~10^9^) contributes strongly to enhance K_α_ emission^24,25^. In these works, the method employed to improve the contrast was to frequency double the pumping pulse^24^. However, at short wavelength, the laser field ponderomotive potential ($\propto I\times {\lambda }^{2}$) as well as the laser heating of the hot electrons is less favorable for efficient x-ray generation. In addition, it implies the spectral change of the initial femtosecond pulse driver with inherent energy efficiency limitations and increased technological difficulties and complexity. With this contrast enhancement method, Eder *et al*.^24^ and Chen *et al*.^25^ showed conversion efficiencies for copper (Cu, Z = 29) K_α_ line as high as 4 × 10^−4^ and 10^−4^ respectively. For Mo K_α_ line and using the same method to enhance the contrast ratio, Fourmaux *et al*.^26^ reported a conversion efficiency which was limited to ~1.5 × 10^−5^. Only recently, as a positive consequence of the development of technological solutions for improving the temporal contrast ratio of high peak-power laser pulses, higher conversion efficiency for Mo K_α_ line (up to 2 × 10^−4^ in 2π sr) was demonstrated at the fundamental wavelength (800 nm) using a multi-TW laser facility with a high contrast ratio^27,28^.

Nonetheless, the role of the contrast ratio remains to be completely elucidated in the high intensity range which motivated the present experimental study. We thus investigate extensively the influence of a high laser pulse contrast ratio for a wide range of pulse intensity on the Mo K_α_ emission efficiency at the fundamental wavelength of 800 nm with a single laser source. This study provides also detailed experimental results for pulsed generation of Mo K_α_ line (17.48 keV) which is suitable for biological and medical applications^6,7^.

## Results

### K_α_ emission versus laser contrast ratio and intensity

**Figure 1:**
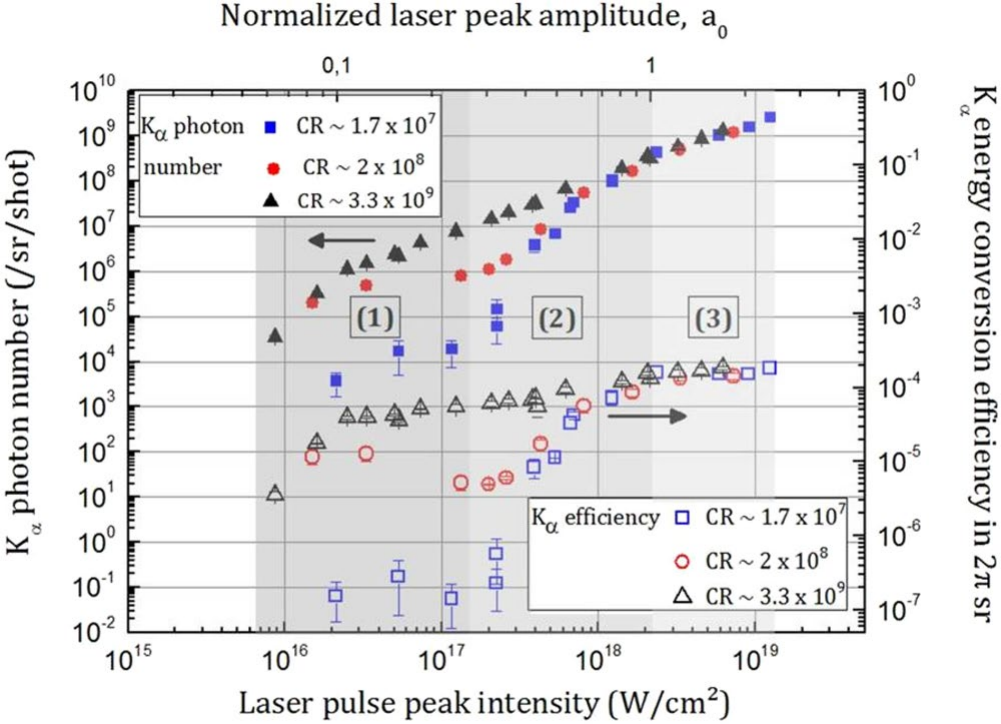
X-ray results versus intensity and contrast ratio. Absolute number of produced K_α_ photons (filled symbols) and K_α_ energy conversion efficiency (open symbols) at the laser incidence angle of 45° as a function of the contrast ratio (CR) and laser peak intensity. The error bar associated with each value is given by the standard deviation calculated from 5 independent measurements of K_α_ photon number/sr/shot. The parameter a_0_ is the dimensionless intensity parameter^12^ given by ${a}_{0}=\frac{e{E}_{L}}{m\omega c}$, where e and m are respectively the electric charge and the electron mass, c the speed of light, ω the laser frequency and E_L_ the laser electric field; the value a_0_ = 1 corresponds to the situation where the electron quiver energy in the laser field allows the electron to have relativistic motion.

Figure 1 shows the evolution of the number of K_α_ photons (${{\rm{N}}}_{{{\rm{K}}}_{{\rm{\alpha }}}})$, emitted per unit of solid angle (sr) per laser shot, for three decades of laser intensity and two decades of contrast ratio respectively ranging from 10^16^–1.3 × 10^19^ W/cm² and 1.7 × 10^7^ to 3.3 × 10^9^. In the same figure, the corresponding K_α_ energy conversion efficiency (η_α_) into 2π sr is also reported. η_α_ is expressed as ${{\rm{\eta }}}_{{\rm{\alpha }}}={10}^{-12}\times \frac{{{\rm{N}}}_{{{\rm{K}}}_{{\rm{\alpha }}}}\cdot {{\rm{E}}}_{{{\rm{K}}}_{{\rm{\alpha }}}}}{{\rm{E}}}$ where ${{\rm{E}}}_{{{\rm{K}}}_{{\rm{\alpha }}}}=17.48\,{\rm{keV}}$, the K_α_ line energy and E the laser pulse energy in mJ.

The results of Fig. 1 shed light on the existence of three intensity regimes regarding the evolution of K_α_ emission. For I < 1.5 × 10^17^ W/cm², which corresponds to the non-relativistic regime 1 (a_0_ < 0.26), K_α_ emission is enhanced by more than two orders of magnitude from the lowest contrast (1.7 × 10^7^) to the highest contrast (3.3 × 10^9^) over the whole intensity range. Besides, this regime is characterized by the different evolution of the K_α_ production for each contrast ratio. A continuous increase of K_α_ emission is measured for the contrast ratio of 3.3 × 10^9^ while a plateau is observed for a specific intensity range (3 × 10^16^ W/cm² < I < 1.5 × 10^17^ W/cm²) for the two lower contrasts 1.7 × 10^7^ and 2 × 10^8^. In the narrow intermediate regime 2 where 1.5 × 10^17^ < I < 2 × 10^18^ W/cm² (0.26 < a_0_ < 1), K_α_ production increases abruptly for the two lower contrast ratios and reaches similar values for I ~ 2 × 10^18^ W/cm², comparable to those obtained with the highest contrast (3.3 × 10^9^). Finally, in the last regime 3 (a_0_ > 1), which corresponds to relativistic intensities (I > 2 × 10^18^ W/cm²), the same K_α_ photon number increase with respect to intensity is measured for all contrast configurations. Thereby, in the range of laser intensity and contrast ratio we explored, K_α_ emission no longer depends on the contrast ratio when working in the relativistic intensity regime.

### Correlation between pre-plasma, contrast ratio and K_α_ emission much below relativistic intensity

In the low intensity range (I < 1.5 × 10^17^ W/cm², regime 1) and for the lower contrast ratio (1.7 × 10^7^), the x-ray production is small, being a factor ~100 below those measured for the two other contrasts (see Fig. 1). We correlate the low photon number and efficiency observed with the lower CR with the existence of a pre-plasma which is formed in this case at very low intensity by the ASE pedestal (approximately from I > 10^16^ W/cm², see *Methods*). It thus has time to expand (L/λ > 1) and to significantly influence the laser energy coupling into the target. We further argue that the difference in K_α_ emission between the contrast ratios can be explained by the plasma density scale length L. Indeed, L is expected to be extremely small for the intermediate (2 × 10^8^) and high contrast ratios (3.3 × 10^9^) because the intensity of the ASE background is too weak to produce a pre-plasma in the low intensity range, strictly until I ≅ 1.1 × 10^17^ W/cm² however for the intermediate contrast ratio configuration (see *Methods*). In these conditions, as the intensity and fluence of the main pulse are huge (approx. 300 J/cm² to 10 kJ/cm² for the regime 1), the target is instantaneously and heavily ionized by the rising edge of the femtosecond pulse which thus imparts the main contribution to the parameter L. Assuming at this short timescale a step-like profile of the plasma gradient and normal skin effect for laser absorption and no hydrodynamic motion, we use the scaling developed by Rozmus *et al*.^29^ to estimate the surface temperature of the plasma layer: ${{\rm{T}}}_{{\rm{e}}}=0.53{{Z}_{eff}}^{1/6}{{\rm{I}}}_{18}^{1/3}{{\rm{\lambda }}}^{-1/6}{{\rm{t}}}^{1/6}\,{\rm{keV}}$ with the intensity I_18_ = I/10^18^ W/cm², λ the wavelength in units of µm and t in fs. Z_eff_ is the effective charge, i.e. the number of electrons that are stripped from the atom in the early beginning of the interaction. Assuming in this intensity range (I ~0.1–1.5 × 10^17^ W/cm²) that the temperature reaches a few hundreds of eV (as calculated after), we suppose multiple ionization of Mo target with ionization of the external electronic layers. Considering the intensity I ≅ 10^17^ W/cm², we obtain: T_e_ ~ 730 eV (with Z_eff_ ≅ 18). Note that this estimation of the surface temperature is little sensitive to the exact knowledge of Z_eff_. The step-like gradient length L then expands with the characteristic ion sound velocity v_s_ expressed by ${{\rm{v}}}_{{\rm{s}}}=\sqrt{\frac{{{\rm{Z}}}_{{\rm{eff}}}{{\rm{k}}}_{{\rm{B}}}{{\rm{T}}}_{{\rm{e}}}}{{{\rm{m}}}_{{\rm{i}}}}}$ m/s where ${{\rm{T}}}_{{\rm{e}}}$ is the plasma temperature in Kelvin, k_B_ is the Boltzmann constant and m_i_ the ion mass. At I ≅ 10^17^ W/cm² and keeping Z_eff_ = 18 (note that different degrees of ionization do not influence significantly the estimation), we calculate: v_s_ ≅ 1.15 × 10^7^ cm/s = 1.15 Å/fs. Therefore, the extension of the plasma density scale length L is calculated to be on the pulse timescale (30 fs) ~3.45 nm, which gives L/λ ≅ 0.004. This value of L/λ parameter is extremely small due to the reduced time for the pre-plasma to expand in the intermediate and high contrast ratio configurations.

**Figure 2:**
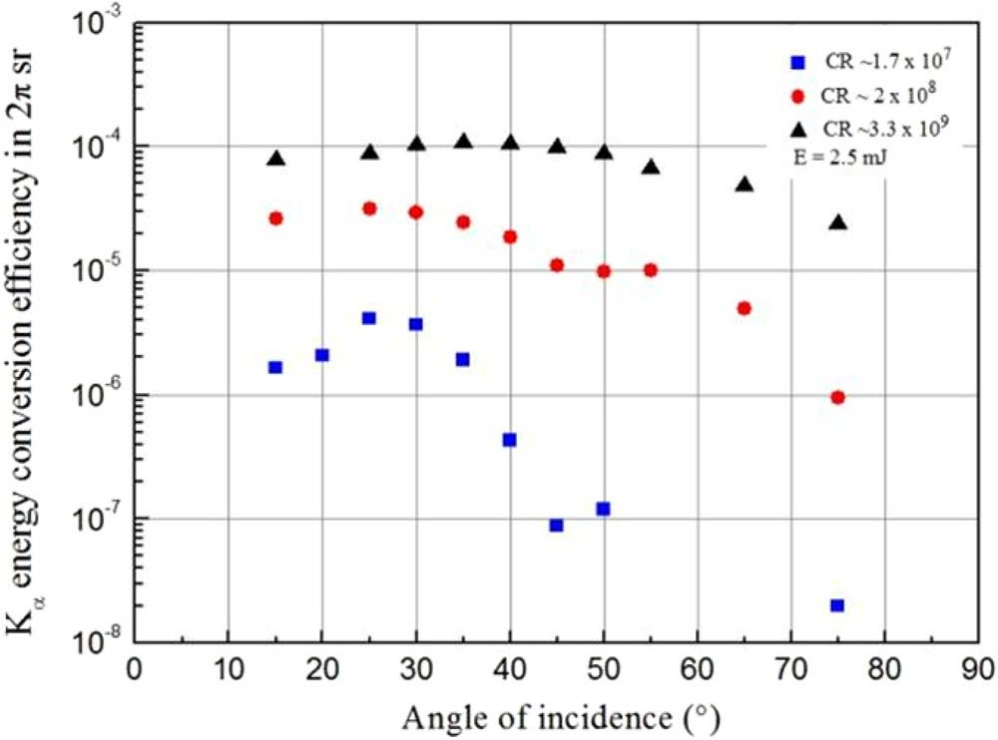
X-ray results at low laser intensity versus angle of incidence. K_α_ energy conversion efficiency as a function of angle of incidence θ for a pulse energy of 2.5 mJ (I = 1.25 × 10^17^ W/cm² at normal incidence) for the three contrast ratios. The angular excursion of the angle of incidence is from ~15° to 75°; it is changed by rotating the target around the vertical axis.

Resonance absorption and vacuum heating are concurrent mechanisms of absorption for irradiances I × λ² < 10^18^ Wµm²/cm². Their relative importance depends especially on the plasma density scale length L/λ^11^. It was also shown that resonance absorption is peaked at some specific angle depending on the value of L/λ. The optimal angle increases when L/λ is reduced below unity^11,12,16^. To test our approach, we performed measurement of K_α_ energy conversion efficiency as a function of the angle of incidence θ and for the three CR. We selected an energy of 2.5 mJ (I ~ 1.25 × 10^17^ W/cm²) corresponding to the range (regime 1) where we observed a huge difference (×100) in terms of x-ray efficiency produced depending on the contrast ratio configuration. The results are presented in Fig. 2.

For the lowest contrast (1.7 × 10^7^), K_α_ energy conversion efficiency is maximal at θ ~ 25° which is in agreement with resonance absorption as the dominant mechanism of absorption. Moreover, in the case of resonance absorption and at a specific angle of incidence ${\rm{\theta }}$, the maximum of absorption is reached for a particular *L* given by^16^: ${L}\approx {\rm{\lambda }}\times (1/2{\rm{\pi }}){(0.8/\sin {\rm{\theta }})}^{3}$. For the angle of θ ~ 25° as found from Fig. 2, the plasma scale length corresponds to L/ λ ~ 1.1. Even if it is extremely difficult to get a clear quantitative estimation of the value of the plasma scale length parameter in practice, this value is consistent with our previous estimation that in the case of lowest contrast ratio configuration an extended pre-plasma (yielding L/λ > 1) is inevitably formed before the main pulse whatever the intensity value in regime 1 (I < 1.5 × 10^17^ W/cm²). In that configuration and regime of intensity for which resonance absorption prevails, we also note that the optimum intensity for K_α_ yield generation is I ~5 × 10^16^ W/cm² followed by a plateau above this intensity (Fig. 1) which is in correct agreement with the numerical estimations of Reich *et al*. (I_opt_ ≅ 9.7 × 10^16^ W/cm²)^14^.

For the intermediate contrast (2 × 10^8^), the maximum of K_α_ conversion efficiency with respect to the angle of incidence (Fig. 2) is less marked and thus the hypothesis of resonance absorption only as the dominant mechanism of laser energy absorption no more holds. For the highest contrast (3.3 × 10^9^), a maximum in K_α_ efficiency is measured for an angle of ~40°. These observations indicate a progressive transition from resonance absorption to vacuum heating when the contrast ratio (enabling step-like L/λ interaction conditions) is enhanced. Indeed, for steep plasma density scale length L/λ ≤ 0.1, vacuum heating is the dominant absorption channel when an optimal absorption is observed for θ ~ 45–50°^11^. This is also in good agreement with our estimation of the L/λ parameter (L/λ ~ 0.004 ≪1) for the highest contrast ratio. In the regime of vacuum heating, strong energy absorption takes place^17^ thus yielding to efficient and large production of x-ray K_α_ photons, as it is evidenced in Fig. 1.

In the case of the intermediate contrast configuration (2 × 10^8^), we assume that vacuum heating is dominant at low intensity while resonance absorption slightly prevails over vacuum heating when a pre-plasma is formed (from I > 1.1 × 10^17^ W/cm², see *Methods*, thus for intensity marginally close to the high limit of regime 1 and to the conditions in which the results of Fig. 2 are obtained). This scenario is in agreement with the evolution of the results observed with the intermediate contrast (2 × 10^8^). Indeed, a weak maximum (for θ ~ 25°) of x-ray energy conversion efficiency is shown at a given angle in Fig. 2 indicating a competition between these two mechanisms of absorption. Moreover, the x-ray photon number is almost equal to the one measured with the high contrast ratio configuration at relatively low intensity when the ASE does not reach a sufficient level to induce a pre-plasma (Fig. 1, I ≪ 10^17^ W/cm²). At higher intensity corresponding to an intensity for which a pre-plasma is formed in the case of the intermediate contrast ratio (from I > 1.1 × 10^17^, see *Methods*), we observe a deviation between the two data curves (x-ray photon number and conversion efficiency, Fig. 1) corresponding to the intermediate and high CR configurations. A decrease in the x-ray efficiency is indeed measured for the intermediate contrast ratio (2 × 10^8^) as well as for the lowest one (1.7 × 10^7^) for intensity of ~1 × 10^17^ W/cm² while it continues to increase (slowly) in the case of the high contrast ratio (3.3 × 10^9^). In these conditions, for which a pre-plasma is formed for the low and intermediate CR configurations and resonance absorption is the main absorption mechanism, we presume that the absorption rate is reduced due to the progressive suppression of favorable conditions for resonance absorption. This is in accordance with recent measurements of laser absorption as a function of intensity which reported a decrease of the fraction of the absorbed laser energy in this intensity range for a contrast ratio of ~10^9^ ^30^. Indeed, Ding *et al*.^31^ pointed out that the absorption rate decreases due to the perturbation of the wave-plasma resonance. In this regime of intensity which is close to the relativistic intensity, the condition of resonance becomes affected by the modulation of the electron mass and the relativistic ponderomotive modulation of the plasma density. Consequently, these effects imply a weaker coupling of the laser energy into the plasma and a reduction of the x-ray conversion efficiency.

Finally, these observations of the evolution of K_α_ conversion efficiency as a function of the contrast ratio in regime 1 point out a progressive transition from resonance absorption to vacuum heating when the contrast ratio is enhanced. Furthermore, our analysis supports the first conclusion that a smaller plasma density scale length contributes highly to enhance K_α_ emission as observed in this non-relativistic regime. We remind also that the results of the K_α_ emission in Fig. 1 are obtained at an angle of incidence of 45° which is chosen to be the optimal angle for vacuum heating mechanism at high contrast ratio (CR > 10^9^). At lower contrast ratio, to optimize K_α_ emission, our results of the K_α_ conversion efficiency as a function of the angle of incidence confirm clearly the need to adapt the working (optimal) angle.

### Progressive importance of laser radiation pressure

At higher intensity (I > 1.5 × 10^17^ W/cm², regime 2), K_α_ production increases abruptly for the low and intermediate contrast ratios before even reaching efficiency values comparable to those obtained with the highest contrast (3.3 × 10^9^) for I = 2 × 10^18^ W/cm². An explanation is given by PIC simulation works^31^. After the initial decrease of absorption efficiency related to the break of resonance conditions (as evidenced before at intensity slightly below 1.5 × 10^17^ W/cm²), they point out that upon further increase of the laser intensity the broadening of the resonance layer may contribute to wave-plasma coupling conditions yielding to a higher absorption rate. However, at high intensity we support that the dominant mechanism to explain this behavior is related to the progressive significance of the laser radiation pressure^32^ which tends to reduce the electron gradient length and thus provides conditions in which L/λ is expected to become sufficiently low to ensure favorable interaction conditions for efficient laser-plasma energy coupling. This explanation is supported by the results obtained by Singh *et al*. in the same intensity regime^30^. The authors measured a redshift of the second harmonic emitted from the critical surface where the laser field is reflected, demonstrating that this surface moves towards the bulk of the target as a result of radiation pressure.

In the regime of high intensity (I > 2 × 10^18^ W/cm², regime 3), a pre-plasma is formed much before the main pulse and expands whatever the contrast ratio (see *Methods*). The leading edge of the main laser pulse penetrates into the pre-plasma before being reflected on the critical density layer ($${{\rm{n}}}_{{\rm{cr}}}=1.1\times {10}^{21}{{\rm{\lambda }}}_{\mu m}^{-2}{{\rm{cm}}}^{-3}$$ corresponding to the free-electron density for which the plasma frequency and the laser frequency are equal). The laser pressure is given by^33^: $${{\rm{P}}}_{{\rm{L}}}=2({\rm{R}}+(1-{\rm{R}})\frac{{\rm{\alpha }}}{2})\frac{{\rm{I}}}{{\rm{c}}}\,\cos \,{\rm{\theta }}$$, with R, the plasma reflectivity, α the fraction of laser light absorbed by the plasma, θ the angle of incidence and I the laser peak intensity. In conditions similar to our experiments for which a pre-plasma is formed, the absorption fraction is measured to be α ~ 0.7 as reported by Singh *et al*.^30^. Thus, to estimate the radiation pressure, we use a reflectivity R ~ 0.3, and for relativistic intensity (I ~ 2 × 10^18^ W/cm²), we obtain P_L_ ~500 Mbar which exceeds considerably the thermal pressure exerted by the plasma ($${{\rm{P}}}_{{\rm{e}}}={{\rm{n}}}_{{\rm{e}}}{{\rm{k}}}_{{\rm{B}}}{{\rm{T}}}_{{\rm{e}}}$$$$).$$ Indeed, using as an approximation the previous scaling of the surface temperature of the plasma (T_e_ ~ 2 keV), we calculate: P_e_ ≅ 5.5 Mbar. Consequently, the pre-plasma is steepened by the optical pressure exerted by the laser and the plasma density scale length L is dramatically reduced, providing conditions for which the absorption of the laser energy (through vacuum heating and J × B heating) becomes highly efficient. To support this explanation, we recall that the results which coincide at an intensity of ~2 × 10^18^ W/cm² show nearly the same K_α_ photon number and K_α_ efficiency obtained whatever the temporal contrast of the pulse. This observation confirms that a phenomenon that takes place at high intensity (a_0_ ~ 1) is progressively cancelling the influence of the pre-plasma formed before the main pulse.

**Figure 3:**
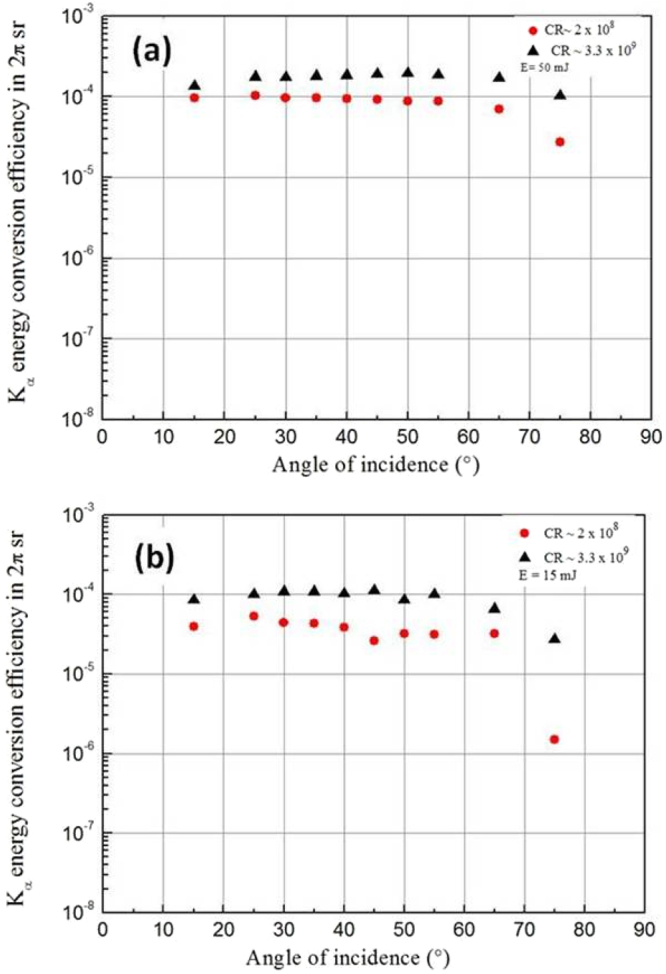
(**a**,**b**) X-ray results at mid and high laser intensity versus angle of incidence. K_α_ energy conversion efficiency as a function of angle of incidence θ for a pulse energy of (**a**) 50 mJ (I = 2.5 × 10^18^ W/cm², relativistic regime) and (**b**) 15 mJ (I = 7.5 × 10^17^ W/cm², below relativistic regime) for the intermediate and high contrast ratios. The angular excursion of the angle of incidence is from ~15° to 75°; it is changed by rotating the target around the vertical axis.

Moreover, in this relativistic regime (a_0_ > 1), the K_α_ production is high and increases similarly with intensity for all contrast ratios. This analysis is in accordance with the progressive significance of relativistic J × B as the dominant mechanism of absorption. In this case, the magnetic component of the Lorentz force becomes significant and relativistic effects dominate. In this intensity regime, accelerated electrons by J × B heating are less diffused by the pre-formed (steepened) plasma and as the magnetic component of the Lorentz force is highly directional, laser energy absorption and thus x-ray generation should become independent of the angle of incidence. This is in full agreement with what we observe in Fig. 3(a) where the x-ray conversion efficiency was studied in the relativistic regime as a function of the incidence angle.

Considering the specific case of the high contrast ratio (3.3 × 10^9^), we also remark that we do not observe a clear transition in the evolution of the K_α_ yield from the non-relativistic to the relativistic regime in Fig. 1. It is consistent with recent works by Gibbon *et al*.^34^ in which they reported that in the relativistic regime, vacuum heating and J × B heating can be described in a unified way as the model becomes insensitive to the details of the absorption mechanism.

Finally, in order to confirm the progressive transition from resonance absorption to vacuum heating as the dominant absorption mechanism in the intermediate intensity regime 2 for the two low contrast ratios, we performed a similar study of the K_α_ energy conversion efficiency for the intermediate and high CR configurations as a function of the angle of incidence for the pulse energy of 15 mJ which corresponds to an intensity at normal incidence of I = 7.5 × 10^17^ W/cm² (Fig. 3(b)). As expected, for the intermediate CR configuration (2 × 10^8^), the x-ray conversion efficiency is high (as compared to the values obtained in Fig. 2) and nearly flat (however still slightly peaked close to θ ≅ 25°) over the large range of variation of the incidence angle. This behavior is consistent with vacuum heating that becomes progressively the dominant absorption channel in the intensity regime 2. Note that the optical pressure calculated at the intensity of I = 7.5 × 10^17^ W/cm² (considering the same approximations as before) amounts to P_L_ ~ 190 Mbar which is sufficiently high to significantly steepen the pre-plasma and to provide favorable interaction conditions for vacuum heating to take place over resonance absorption. For the high CR configuration (3.3 × 10^9^), the x-ray conversion efficiency is also high, almost flat (nonetheless with a slightly marked maximum at θ = 45°) with respect to the large excursion of variation of the incidence angle. This behavior is also in agreement with the progressive transition from vacuum heating to J × B heating in this intensity range.

## Discussion

Following our measurements and the proposed interpretation described above, we now recall the main results obtained. Firstly, when the laser intensity is below the relativistic intensity (I < 2 × 10^18^ W/cm²), we observe the importance of the contrast ratio parameter CR which, together with the laser intensity, controls the characteristics of the K_α_ emission. In this intensity regime, it was shown that resonance absorption or vacuum heating mechanism can take place depending on the specific values of contrast ratio and laser peak intensity. In particular, high contrast ratios (sufficiently high to not induce a pre-plasma before energy deposition) are desirable for favoring vacuum heating mechanism and producing high K_α_ photon number and energy conversion efficiency in our geometrical experimental configuration (45° laser angle incidence on the target). Interestingly, we also set in evidence that extremely different K_α_ photon number and energy conversion efficiency are obtained, even when similar pre-plasma characteristics are induced, which underlines the driving role of the laser peak intensity. As an example, let us consider the two following interaction conditions (CR = 1.7 × 10^7^ and I ~ 10^16^ W/cm² (1) and CR = 2 × 10^8^ and I ~ 10^17^ W/cm² (2)), which have similar pre-pulse intensity thus presumably generating similar pre-plasma density scale length (L/λ) before the main femtosecond pulse. Note that L/λ is very small (L/λ ≪1) in these two operating conditions since the ASE fluence is below the ablation threshold fluence of Mo (see *Methods*). However, the K_α_ photon number and energy conversion efficiency are approximately two decades higher in the case (2) (see Fig. 1) which corresponds to high intensity conditions. Indeed, the laser peak intensity strongly influences the pre-plasma density scale length and the absorption properties (and related efficiency) during the crucial (and most important) phase of the interaction, i.e. during the energy deposition corresponding to the main femtosecond pulse. Considering this particular case (for which we set in evidence the predominance of resonance absorption like in the case (1), see section “Results”), we hypothesize that the laser intensity is getting close to the optimum intensity given by Reich *et al*.^14^ which is for molybdenum I_opt_ ≅ 9.7 × 10^16^ W/cm². In this configuration, hot electron energy distribution favoring a good energy conversion transfer is presumably obtained to generate efficiently K_α_ lines.

At high intensity in relativistic regime (I > 2 × 10^18^ W/cm²), we demonstrate that K_α_ emission is independent of the value of the contrast ratio and continues to increase slowly. In the same intensity range, K_α_ conversion efficiency tends to form a plateau, which was not reported before. At relativistic intensities, the pre-formed plasma is considerably reduced due to the optical pressure exerted by the laser. Therefore, the interaction conditions for the laser energy absorption become independent of the temporal contrast ratio (at least in the CR range we explored from CR > 10^7^). Moreover, they allow efficient laser energy coupling (through relativistic J × B heating absorption mechanism) into the target. This is favorable to the achievement of high and similar efficiency of K_α_ emission for all configurations of contrast ratio.

In the relativistic regime, for which a plateau of K_α_ conversion efficiency is observed, we also believe that there is a competition between two effects: (i) the reabsorption of K_α_ photons induced deeply in the massive target and (ii) the K-shell ionization cross section which depends on the energy of the hot electrons generating K_α_ lines. An estimate of the hot electron temperature which characterizes the energy distribution of the hot electron population at high intensity is given by the scaling of temperature based on the ponderomotive relativistic force^18,19,35^: ${{\rm{T}}}_{{\rm{h}}}\approx 511[{(1+0.73{{\rm{I}}}_{18}{{{\rm{\lambda }}}_{\mu m}}^{2})}^{1/2}-1]\,{\rm{keV}}$. For relativistic intensity (I > 2 × 10^18^ W/cm²), we have T_h_ ≥ 200 keV. In this energy range, the K-shell ionization cross section tends to re-increase due to relativistic effects^36,37^ and to be close or even superior to its first maximum at low energy for high Z elements. Note that this first maximum is close to the K-shell ionization energy which is for Mo, E_K_ ≅ 20.1 keV^38^. However, in the same intensity range, an energetic electron may induce K_α_ emission deeper in the target. For hot electron temperature in the range of a few hundreds of keV to a few MeV, the maximum range R of the energetic electrons in Mo target can be estimated by^39^: ${\rm{R}}({\rm{cm}})=0.412{{\rm{E}}}_{{\rm{h}}}^{1.265-0.0954{{\rm{lnE}}}_{{\rm{h}}}}/{\rm{\rho }}$, with E_h_ the energy of the electrons (in MeV) and ρ the Mo mass density (ρ = 10.28 g/cm^3^). This yields to R ~ 17–400 µm for hot electrons 0.1 MeV < E_h_ < 1 MeV. Using the mass attenuation coefficient µ/ρ for x-ray K_α_ photons in Mo (µ/ρ ≅ 20 cm²/g yielding to an attenuation coefficient µ ≅ 203.4 cm^−1^)^40^, we calculate the absorption length in Mo for which the K_α_ photon flux is divided by the factor *e* to be l_1/e_ ≅ 50 µm, which appears to be comparable to the distance of penetration of the hot electrons. In particular, for the electron energy of ~210 keV (reached for I ~2.15 × 10^18^ W/cm², using the previous scaling equation), the maximum distance of penetration of hot electrons in the target amounts to ~50 µm. These estimates allow us to set into evidence that the two competing effects, K_α_ photon reabsorption and the increase of the K-shell ionization cross section, occur in the intensity range explored in regime 3. This consistently explains the formation of the plateau of K_α_ efficiency in the relativistic regime. Furthermore, the K_α_ photon production continues to rise due to the increase of the laser intensity (note that the energy was the parameter to scale up the intensity in this experiment). Presumably, but with an importance to be better evaluated, the increase of K-shell ionization cross section for high energy electrons, which is progressively becoming sufficiently high to overcome the photon K_α_ losses related to photon reabsorption, also supports the continuous increase of the K_α_ photon number in the relativistic regime.

## Conclusion

In summary, we performed a thorough experimental investigation of the impact of temporal pulse contrast ratio and laser intensity on K_α_ emission produced from the interaction of an intense femtosecond laser with a thick molybdenum target. The study is carried out over wide ranges of contrast ratio 1.7 × 10^7^–3.3 × 10^9^ and intensity 10^16^–1.3 × 10^19^ W/cm² with a single experimental setup. The obtained results of K_α_ photon number and energy conversion efficiency are full of information concerning the physics of ultrahigh and femtosecond laser solid interaction. From our experimental results and absorption modeling found in literature, we point out the main absorption mechanisms that occur for each contrast ratio configuration and intensity range considered in this study. Additional experimental results where the K_α_ energy conversion efficiency is studied as a function of the angle of incidence show clearly different absorption mechanisms for each contrast configuration and intensity condition. In the non-relativistic intensity regime, we confirm experimentally that the plasma density scale length L/λ, which depends on the contrast ratio of the driving laser pulse, and the laser peak intensity are both crucial parameters which control the efficiency of K_α_ generation.

In the relativistic regime, we obtain an original result stating that the K_α_ yield is observed to be independent of the value of contrast ratio in the studied range of contrast (CR > 10^7^). Furthermore, no saturation of K_α_ photon number (which indeed continues to increase with intensity) is seen and K_α_ energy conversion efficiency shows a slow increase with intensity before to form a plateau in the highest intensity range explored in the present work. These investigations allow us to confirm that high K_α_ conversion efficiency (~2 × 10^−4^ in 2π sr) can be reached from laser-produced plasma with a thick molybdenum target. Moreover, the demonstration of a K_α_ source photon flux of 2 × 10^10^ photons/sr/s shows the great potential as a secondary source for further applications like phase contrast imaging and time-resolved x-ray absorption spectroscopy or x-ray diffraction measurements.

## Methods

### Experimental arrangement for x-ray generation

Experiments were performed at ASUR facility in LP3 laboratory^41,42^. The 20 TW line *(Amplitude Technologies)* based on Ti:Sa technology and chirped pulse amplification technique delivers an output beam at 800 nm with super-Gaussian spatial profile and energy up to 300 mJ on target with rms energy stability of ~2.5% at 10 Hz repetition rate. The pulse duration is ~30 fs Full Width at Half Maximum (FWHM) as measured using a self-referenced spectral interferometric device (Wizzler, *Fastlite*). The p-polarized beam is focused on a 6 mm thick polished (roughness < 1 µm) rotating molybdenum disk with a f/4 silver-coated off-axis parabolic mirror (OAP). A 1 mm thick fused silica wafer with broadband antireflection coating protects the OAP from debris produced during laser-target interaction. It is positioned perpendicular to the laser beam and as close as possible to the OAP to minimize the development of nonlinear effects. Considering the group velocity dispersion of fused silica (361 fs²/cm @ 800 nm), note that the pulse duration is not affected. Nevertheless, even if we cannot completely rule out the development of nonlinear effects at the maximum intensity used in the experiments (1.3 × 10^19^ W/cm²), we assume that they do not significantly affect the laser characteristics in the focal plane. Moreover, the distortions of the incident laser wavefront are corrected using a deformable mirror (*Imagine Optic)* which takes into account the aberrations induced by all the optical components present on the path of the beam until the target. The focal spot is then imaged using reflection from two uncoated plane optical substrates positioned at 45° and a relay imaging system coupled to a beam analyzer detector with a magnification factor of 8. The focal spot diameter is measured at ~6 µm (FWHM). Considering the real 2D spatial profile of the beam at the focal point, the pulse energy, the pulse duration and neglecting the spatio-temporal coupling of the beam, the maximum peak intensity reaches ~1.3 × 10^19^ W/cm² at normal incidence. Note that the laser intensity is varied by changing the pulse energy in our experiments by means of a half-wave plate (λ/2) and a polarizer located in the final power amplifier of the laser chain.

### Contrast ratio characterization and modification

The pre-pulses in the nanosecond time range are detected before the compressor with a fast photodiode (250 ps rise time) coupled to a 3.5 GHz oscilloscope and a set of calibrated neutral density filters at 800 nm. To characterize the temporal contrast associated with ASE or pulses replicas on a time scale up to 0.5 ns prior to the main pulse, we use a third order autocorrelator (Sequoia, *Amplitude Technologies*).

**Figure 4:**
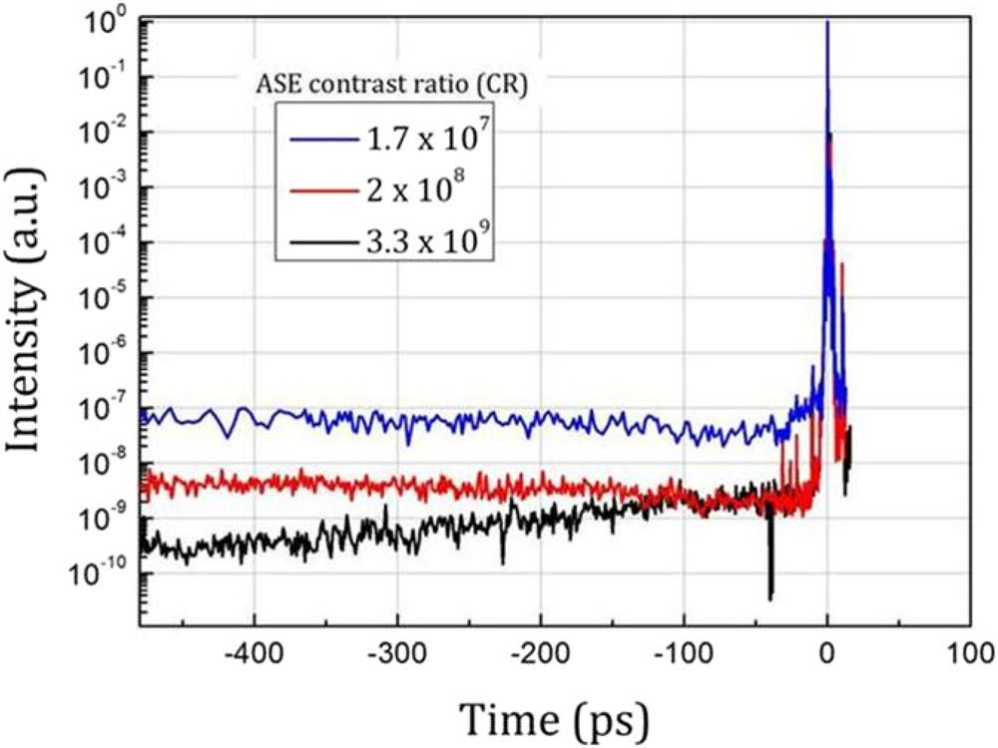
Measurement of contrast ratio.Third-order autocorrelation measurement of the laser pulse measured at picosecond time scale and for different configurations of temporal contrast ratio.

In the laser chain, a multipass amplifier (Booster amplifier) is inserted in between the oscillator and the stretcher. The non-stretched beam is amplified up to a few µJ per pulse and then it is temporally cleaned by a saturable absorber (SA) that removes the residual ASE background of the pre-amplified beam as well as the oscillator pulse replicas. The main objective of this preamplifier is to keep the amplified spontaneous emission (ASE) level of the amplified beam as low as possible by injecting a clean and high-energy seed-pulse in the regenerative amplifier located after the stretcher. Figure 4 (red plot) shows the temporal profile of the amplified and compressed beam obtained by third order autocorrelation in this intermediate contrast ratio configuration. In the present work, we vary the temporal contrast in a controlled way to get three different temporal contrast configurations:

- We decrease the temporal contrast (low contrast ratio configuration) by removing the initial SA and reducing the pump energy in the Booster amplifier. The regenerative amplifier is thus seeded by a train of pulse with moderate energy and the pumping level of the regenerative amplifier is increased to correctly saturate the amplifier. The ASE level is therefore increased by one order of magnitude (blue curve in Fig. 4).
- We keep the initial SA in the Booster amplifier and the nominal pump energy in the Booster and we further improve the temporal contrast by inserting two extra SA in between preamplifiers. In this high contrast ratio configuration, the ASE level is decreased progressively from −100 ps to −480 ps (black curve in Fig. 4). However, for time ranging from −100 ps to the main pulse, the ASE level is unchanged as compared to the intermediate contrast configuration.

For simplicity purpose, we distinguish the three different contrast ratio configurations by their ASE contrast $CR\,({C}{\rm{R}}={{\rm{I}}}_{{\rm{peak}}}/{{\rm{I}}}_{{\rm{ASE}}})$ values at −480 ps respectively 1.7 × 10^7^, 2 × 10^8^ and 3.3 × 10^9^ for low, intermediate and high CR configurations.

For the different temporal contrast configurations described above, we also measured three pre-pulses in the ns range (at −9.8, −8.3 and −5.5 ns) with the fast photodiode. The pre-pulse at −9.8 ns, related to the leakage of optical elements sensitive to polarization in the regenerative amplifier, was minimized (CR > 8 × 10^9^) by careful management of the extraction Pockels cells and of the delay of the amplifier pumps. The contrast ratio of the two other pre-pulses, issued from pulse scattering induced by defects in the amplification crystals or mirrors in amplifiers^43^, was measured with the photodiode equal to 8 × 10^7^. Actually, the intensity contrast ratio related to those pre-pulses is much higher by several orders of magnitude since the divergence and dispersion of those pre-pulses are strongly deteriorated with respect to those of the main femtosecond pulse^43^.

Finally, the pre-pulses do not contain enough energy to yield a level of intensity or fluence sufficient to ionize or damage the target prior the arrival of the main pulse, even at the maximum intensity whatever the contrast configuration.

In our operating conditions, mainly the nanosecond pedestal due to amplified spontaneous emission (ASE) can thus create a pre-plasma with density scale length L/λ able to modify the absorption of the laser pulse. Using a fast photodiode, we found that ASE pedestal starts about τ_ASE_ ~ 5 ns before the main pulse. Molybdenum is characterized by I_ionization-threshold_ ~ 10^12^ W/cm² and an ablation threshold F_ablation-threshold_ ~ 2.7 J/cm² for the pulse duration of ~5 ns as deduced from reference^44^. Considering the maximum intensity reached in our experiments (I ~ 1.3 × 10^19^ W/cm²), the intensity of the ASE pedestal (I_ASE_ ~ I_peak_/CR) is below the target ionization threshold whatever the contrast ratio. Thus a pre-plasma can be formed only when the ASE fluence is sufficiently high to vaporize the target. In the studied intensity range, the formation of a pre-plasma takes place approximately from low intensity (I > 1 × 10^16^ W/cm²) for the lowest CR configuration (1.7 × 10^7^) and from intermediate (I > 1.1 × 10^17^ W/cm²) and high (I > 1.8 × 10^18^ W/cm²) intensity for the intermediate 2 × 10^8^ and high 3.3 × 10^9^ CR configurations respectively.

### Measurement of K_α_ photon number

The x-ray spectra are measured using a direct-detection back-illuminated CCD camera (*PIXIS-XB*, *Princeton Instruments*) cooled down to −40 °C. The detector has a 16 bit dynamic range and it is equipped with a chip size of 1024 × 1024 pixels of 13 µm × 13 µm pixel size. The camera is located at 92 cm from the target, in the horizontal plane defined by the target normal and the incoming laser beam (set initially at 45° angle of incidence). The CCD is positioned at an angle of 45° with respect to the normal of the target front side. In all experiments, the position of the CCD x-ray detector is kept unchanged. Note that K_α_ emission is isotropic for p-polarized laser excitation pulse in the considered intensity range^45^. The x-ray detector is also protected from charged particles by a couple of magnets positioned in the interaction chamber at 6 cm from the target. In order to avoid unwanted background radiation that can reach the camera, the latter is connected to the interaction chamber via a tube under vacuum (pressure ~30 mbar) and a 800 µm-thick Beryllium window. Moreover, a set of three lead apertures is installed in the tube and the camera is protected with a shielding made of lead of 3 mm thickness. In order to avoid pile-up problems at high laser pulse energy due to the summation of the energy of more than one photon within one pixel, a set of metallic filters with adapted material and thickness are used. In our case, two filters are put close to the x-ray source. They consist on Aluminum and Titanium with 1 mm and 125 µm thicknesses respectively.

The camera is used as a dispersionless spectrometer operating in photon counting regime^46^. Photon counting mode consists on the detection of a single photon per pixel and per shot. This allows us to measure the source spectrum with a single laser shot (high intensity regime) or with the accumulation of few laser shots (low intensity regime) to get good signal to noise ratio and accuracy in this regime.

**Figure 5:**
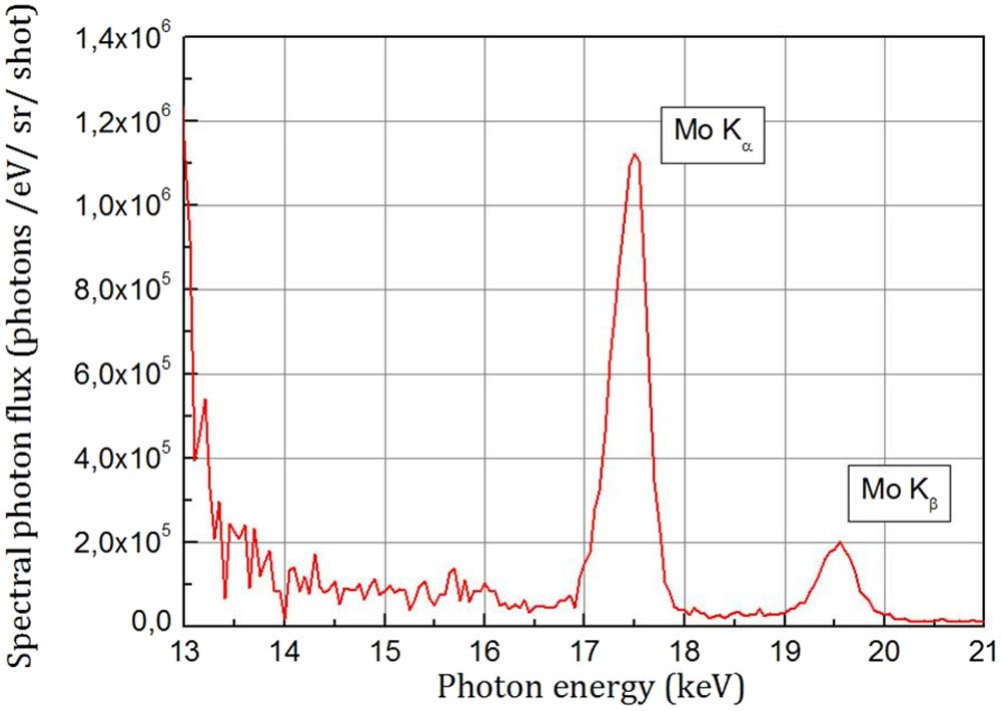
Absolute energy spectrum of the Mo x-ray emission obtained with the help of the X-CCD camera for the laser intensity I = 2 × 10^18^ W/cm² and for the contrast ratio configuration 3.3 × 10^9^.

To reconstruct the spectrum of the x-ray source, a home-developed software and a Co^57^ radioactive calibration source allow us to obtain absolute x-ray spectra within 2–20 keV photon energy range. We take into account the quantum efficiency of the detector which depends on the photon energy, the transmission of the Beryllium window and of the metallic filters, the solid angle of detection and the number of laser shots. A typical spectrum with well resolved Mo K_α_ and K_β_ lines at 17.48 keV and 19.6 keV respectively, is shown in Fig. 5.

Due to the limitations of our calculation and of the spectral resolution of our detector, the precision of the spectrum reconstruction is ~200 eV^42^. In our work, the K_α_ photon number corresponds to the number of photons counted in the photon energy range 17–18 keV. At low intensity, the spectrum is measured with the accumulation of few laser shots for enhanced accuracy. In this case, the calculation takes into account the number of laser shots to determine an averaged K_α_ Mo spectrum. From the spectrum obtained for every configuration of laser intensity and contrast ratio, we further deduce the K_α_ photon number/sr/shot. This complete procedure was repeated five times for every configuration defining the full set of data and error bars in Fig. 1.
